# Shear strength of light weight concrete elements model based on deep neural network and COVID-19 optimization

**DOI:** 10.1038/s41598-025-20538-0

**Published:** 2026-05-19

**Authors:** Mohamed A. Shamseldin, Ahmed Farouk Deifalla, Denise-Penelope N. Kontoni, Medhat Araby

**Affiliations:** 1 Mechatronics Engineering Department, Faculty of Engineering, Future University In Egypt, New Cairo, Egypt; 2 Structural Engineering and Construction Management Department, Faculty of Engineering, Future University in Egypt, New Cairo, Egypt; 3 Department of Civil Engineering, School of Engineering, University of the Peloponnese, Patras, GR-26334 Greece; 4 School of Science and Technology, Hellenic Open University, Patras, GR-26335 Greece; 5 FBB Motors Investment Co., HONGQI authorized dealer, Dubai, United Arab Emirates

**Keywords:** COVID-19 optimization, Deep neural network, Shear strength, Light weight concrete elements, Engineering, Materials science

## Abstract

Predicting the shear strength of concrete elements is a complex challenge influenced by numerous factors, with the type of concrete playing a decisive role in structural performance. Lightweight concrete, offering a superior strength-to-weight ratio and improved thermal properties compared to conventional weight concrete, has gained increasing adoption in structural applications. This study proposes a deep neural network (DNN) model optimized using the COVID-19 optimization algorithm to predict the shear strength of lightweight concrete elements. The optimization process determines the optimal initial weights and biases of the DNN, enhancing convergence and accuracy. The proposed model was evaluated against three international design codes—ACI, EC2, and JSCE—using experimental datasets. Results show that the COVID-19–optimized DNN closely simulates and tracks actual shear strength values, even in highly nonlinear data regions (e.g., samples 90–120), where traditional models produce larger deviations. Quantitatively, the proposed DNN achieved the lowest average error (0.692) compared to the higher errors from ACI, EC2, and JSCE models. These findings demonstrate the model’s superior predictive capability and its potential to enhance design accuracy for lightweight concrete structures.

## Introduction

Reinforced concrete (RC) elements are subjected to flexure, shear, or axial forces^1–4^. The flexure and axial design of RC elements is well established, while much less information about the shear design of RC elements. Design codes worldwide treat shear design differently. The previous studies showed a comparison between selected design codes containing the ACI, the EC2, and the JSCE^1–3^. It is clear that significant difference between design codes in model parameters and assumptions.

Numerous factors and processes control the shear failure of reinforced concrete elements. Furthermore, shear fractures can be caused by shear forces or flexure fractures caused by bending moments^5–10^. Further investigation is necessary to fully understand the behaviour of LC components under shear because it is complex on multiple levels^11]– [12^.

Stress distribution becomes complicated after the initial cracking initiation and is influenced by a number of factors and mechanisms. Late in the 1990s, Committee 445 of ASCE-ACI^13–19^, the following significant shear transfer mechanisms have been reported: (1) shear in the element’s uncracked compression zone; (2) shear in the interface where cracks are forming because of surface roughness and aggregate interlock; (3) the longitudinal reinforcement’s dowel action; (4) residual tensile stresses across inclined cracks; and (5) the arch mechanism^19–25^.

The use of lightweight concrete (LC) increases the problem’s complexity. Despite this, LC is becoming more widely accepted worldwide^26–30^. In comparison to normal-weight concrete (NC), it is substantially lighter, has superior insulation, and is more ductile^30–33^. Furthermore, there was a substantial breakthrough in the technology of concrete manufacturing, which led to a significant evolution in the performance of LC^33–40^. Furthermore, as LC becomes more popular, new shear design guidelines are required^41–44^. In contrast to the NC, where the cracks are smooth as they flow through the aggregate, the cracking in LC is different. Consequently, the aggregate interlock mechanism’s shear resistance is weakened^45]– [46^.

A ground-breaking innovation in the field of artificial intelligence (AI) is deep neural networks (DNNs). These networks can describe intricate non-linear connections because they feature numerous hidden layers between the input and output levels. DNNs have revolutionized numerous fields, including natural language processing, computer vision, and speech recognition, by being able to recognize and comprehend complex patterns^47–49^.

A neural network is fundamentally made to take in inputs, carry out calculations, and produce results to address problems in the real world. DNNs depend heavily on the sequential data flow within the network. A DNN may have many more hidden layers than a standard neural network; some networks may have up to 1000 layers. Compared to conventional machine learning models, DNNs can handle more complicated problems and give better outcomes because of their depth^50–52^.

Deep learning networks have been used in a variety of fields, such as text analysis, time series analysis, and dynamic image analysis. The mapping of images to user names in social networks is a prominent application. Additionally, deep learning has demonstrated its adaptability in problems involving natural language processing by enabling the development of descriptive sentences for photographs^53^.

Each layer in deep neural networks is made up of nodes that convert inputs into outputs. As the network develops, the weights and biases associated with each layer change, enabling more intricate transformations. Inputs are passed via layers throughout the forward propagation process in order to activate nodes and eventually arrive at the output layer^54^.

In a neural network, the credit assignment path (CAP) symbolizes the chain of conversions from input to output. CAPs create a causal link between the input and the output, revealing how the network makes decisions. Given that the output layer is taken into account, the CAP depth for feed-forward neural networks is equal to the number of hidden layers plus one. The CAP depth in recurrent neural networks may be infinite because signals might pass through layers numerous times^55^.

Although it can be difficult to distinguish between deep learning and shallow learning, it is generally agreed that deep learning networks have a CAP depth of at least two. Simple patterns can be recognized using shallow networks, which have fewer layers. However, deep neural networks are required as pattern complexity rises. Deep networks are excellent at dissecting complicated patterns into simpler ones, which enables them to recognize complex structures like human faces.

DNN training can be difficult because of problems like vanishing or exploding gradients. When using backpropagation to train networks, which modifies weights and biases in order to reduce the cost function, certain issues can occur. However, newer developments like Deep Belief Networks (DBNs) and Restricted Boltzmann Machines (RBMs) have solved these problems.

Accurate prediction of the shear strength of reinforced concrete (RC) elements remains a long-standing challenge due to the multi-factor interactions among material properties, geometry, and reinforcement details. While design provisions (e.g., ACI, EC2, JSCE) offer conservative equations, their generalized assumptions can misrepresent cases with pronounced material heterogeneity—such as lightweight concrete (LWC) where reduced unit weight, different aggregate properties, and altered fracture mechanisms modify shear transfer and size effects. Consequently, data-driven models have gained traction as they can capture nonlinearities that are difficult to encode analytically^54–56^.

Over the past few years, a growing body of studies has applied machine learning (ML) to predict shear capacity of RC members, using algorithms such as artificial neural networks (ANN), random forests (RF), M5 model trees, extreme learning machines (ELM), adaptive neuro-fuzzy systems (ANFIS), XGBoost, and gene/expression-based evolutionary models (GP/GEP). Across beam, joint, and slab problems, these approaches generally exceed the accuracy of closed-form code equations and legacy regressions. Examples include RF/M5/ELM models for shear of FRP-RC beams, ANN/ANFIS/XGBoost for corroded RC beams, and GEP/GP variants for beam shear and joint shear, often reporting lower RMSE/MAPE and higher R^2^ than code predictions^56–58^.

Specific to LWC, dedicated ML studies are comparatively fewer but are emerging. Recent works trained multiple ML algorithms on curated LWC (and mixed LWC/normal-weight) beam databases; they reported noticeable gains over design codes and produced data-driven insights on influential variables, yet highlighted sensitivity to data distribution and feature selection. For example, studies using ensembles, ANN, evolutionary polynomial regression, and GP on datasets containing LWC specimens achieved improved accuracy versus code equations and proposed interpretable expressions—but with limits in external validation and generalizability when the nonlinear regime intensifies^58–60^.

Despite this progress, several research gaps persist for LWC shear prediction. The first gap,

LWC-focused datasets and benchmarking. Many ML studies either pool LWC with normal-weight concrete or analyze specialized conditions (e.g., corrosion, FRCM strengthening), making LWC-only benchmarking against multiple codes and ML baselines relatively scarce.

The second gap, deep learning with principled optimization. While ANNs/ELMs appear frequently, comparatively few LWC studies deploy deep neural networks (DNNs) with systematic metaheuristic optimization of initial weights/biases to improve convergence and reduce local-minimum sensitivity. Most rely on default training heuristics or conventional evolutionary models (GP/GEP).

The third gap, robust evaluation under strong nonlinearity. Prior works often report average metrics; fewer explicitly analyze localized high-nonlinearity regions (e.g., specific specimen index ranges) where code-based models and standard ML degrade, or provide fine-grained error profiling.

The fourth gap, transparent comparisons to design codes. Head-to-head evaluations against ACI/EC2/JSCE for LWC beams exist but are not yet routine across diverse ML architectures with uniform metrics and error breakdowns.

This study addresses these gaps by developing a COVID-19–optimized deep neural network (DNN) tailored to predict the shear strength of LWC elements. The COVID-19 optimization algorithm is used to select the DNN’s initial weights and biases, aiming to accelerate training and enhance generalization. We conduct systematic benchmarking against ACI, EC2, and JSCE equations and ML baselines, reporting standard metrics (e.g., R^2^, RMSE, MAPE) and localized error analyses in highly nonlinear data regions. As a result, the proposed model demonstrates consistently lower average error than code-based models and improved tracking of the actual response in challenging segments, highlighting its potential as a practical, data-driven complement to existing design provisions.

## DNN model development

Neural networks called Deep Belief Networks (DBNs) are made up of a stack of RBM (restricted Boltzmann machine) layers that are trained independently one after the other to create progressively abstract representations of the inputs in higher layers.

Probably the first multilayer learning machine motivated by statistical mechanics was the Boltzmann network (or machine). The visible unit’s layer and the hidden unit’s layer are the two layers that make up the network. The network is made up of stochastic neurons, employs symmetric and bidirectional connections between neurons, and lacks connections between neurons in the same layer. Both directions have the same weights.

**Fig. 1:**
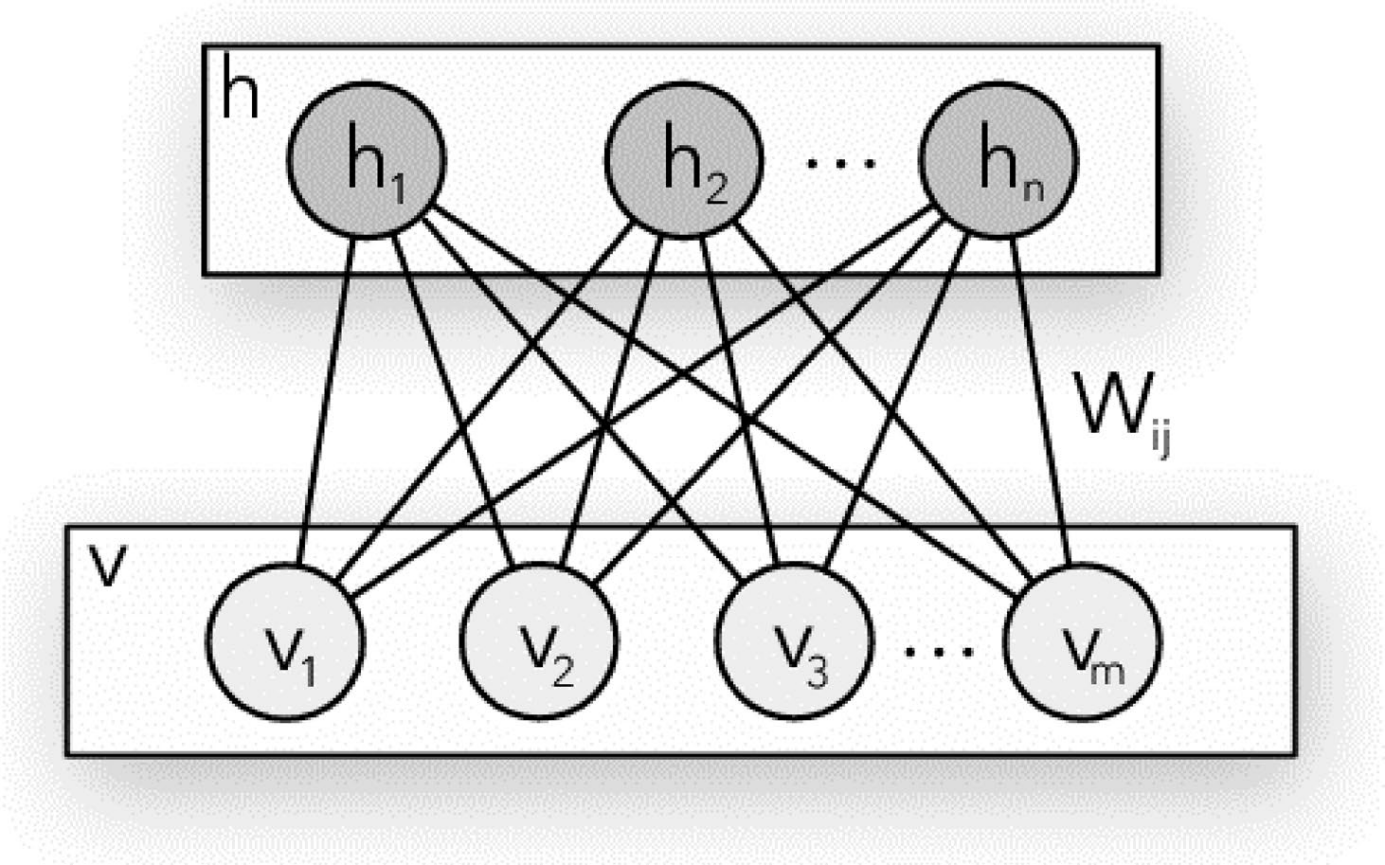
RBM Architecture.

Every RBM contains a visible input layer and a hidden layer of stochastic binary units, as depicted in Fig. 1. A weight matrix connects the visible and hidden levels, and there are no connections between units in the same layer. There are two possible paths for signal propagation: reconstruction, in which hidden activations spread to visible units, and recognition, in which visible activations spread to the hidden units. Both the recognition and the reconstruction processes use the same weight matrix (transposed). By the use of a technique known as contrastive divergence (CD), which minimizes the difference between the original input and its reconstruction (i.e., reconstruction error).

The RBM can be trained to produce the input patterns it receives with a high probability by using the weights. The weights of a deep neural network can be initialized using the RBM pre-training process of a DBN. Back-propagating error derivatives can then be used to discriminatively fine-tune the network. A conventional neural network adopts the DBN’s “recognition” weights as its own weights. To fine-tune the generatively trained parameters, a hybrid training technique can be applied when the RBM models the combined distribution of observable data and class labels.

### RBM mathematical approach

A restricted Boltzmann machine (RBM) is an undirected graphical model with two layers of units: visible (observed) and hidden (latent). The absence of connections within each layer means the visible and hidden units form a bipartite graph. Let v denote the column vector representing the states of the visible units and $\:h$ the column vector for the hidden units. For every combination of visible and hidden state vectors, a binary RBM assigns an energy value $\:E(v,h)$ based on the model parameters. This energy function governs the joint probability distribution of $\:v$ and $\:h$ and is central to the RBM’s learning process.

1 $$E(v,h)= - {b^T}v - {c^T}h - {v^T}{\mathrm{Wh}}$$

In this case, b is a visible unit bias (column) vector, c is a hidden unit bias (column) vector, and W is the matrix of visible/hidden connection weights. The likelihood of a specific configuration of the visible and hidden units, expressed in terms of the configuration’s energy, is provided by

2 $$P(v,h)=\frac{{{e^{ - E(v,h)}}}}{Z}$$

where the partition function is denoted by the normalisation factor *Z* =∑_*v, h*_ *e*−*E*(*v, h*)). Since the visible units are tentatively sovereign given the hidden unit states and vice versa, we may obtain simple exact formulae for *P*(*v*|*h*) and *P*(*h*|*v*) due to the lack of direct links inside each layer. In the instance of binary units, the conditions are provided by: (for a derivation, see Bengio [2009]).

3 $$P(h|v)=\sigma (c+{W^T}v)$$

4 $$P(h|v)=\sigma (b+Wh)$$

Where σ(∎) is the firing rule of the network neurons. The sigmoid function σ(∎) may be expressed by the following

5 $$\sigma {(\Delta E)_j}{\kern 1pt} j=\frac{1}{{1+\exp ( - \Delta {E_j}/T)}}~~~$$

Where ∆*E*j = *net*j = *the sum of j*th *neuron inputs*; and the variable (T) is considered the network temperature as it controls the energy (∆*E*j) rate.

Ideally, we would follow the gradient of the maximum likelihood of the data in order to train an RBM. This results in the following update algorithm for an arbitrary model parameter θ, which is based on expectations of the energy gradients over the data distribution and the model distribution.

6 $$\:\varDelta\:\theta\:\alpha\:{\left(\frac{\partial\:E}{\partial\:\theta\:}\right)}_{data}-\:{\left(\frac{\partial\:E}{\partial\:\theta\:}\right)}_{model}$$

Specifically, the visible-hidden weights’ maximum likelihood update rule is:

7 $$\Delta W{\kern 1pt} \alpha {({v_i}{h_j})_{data}} - {({v_i}{h_j})_{model}}$$

The frequency that the visible unit *v*i and the hidden unit *h*j are on together in the training set is the expectation (*v*i *h*j) *data*, and that same expectation under the distribution given by the model is (*v*i *v*j) *model*. We are unfortunately obliged to apply an approximation because the term (. ) *model* takes exponential time to compute exactly. Contrastive divergence (CD) can be used to train RBMs since they are situated at the nexus of expert model output and Boltzmann machines. The following becomes the new updating rule:

8 $$\Delta W{\kern 1pt} \alpha {({v_i}{h_j})_{data}} - {({v_i}{h_j})_l}$$

The expectation, denoted by (*v*i *h*j )*l*, pertains to the sample distribution that results from executing the Gibbs sampler for a full step, starting with the data and defined by utilising Eqs. 3 and 4.

### RBM training algorithm

1. Take the training dataset, and set the states of the visible units to the training dataset.
2. *Positive Phase*: Update all the hidden units in parallel using Eq. 3. Compute positive statistics for (*v*i *h*j)*data* which is *P*(*h*j = 1|*v*).
3. *Negative Phase*: Reconstruct visible units using Eq. 4. Compute positive statistics.

for (*v*i*h*j )*l* which is *P*(*v*i = 1|*h*).

4. *Updating weights*: Updating weights W` ij based on the old weights Wij using W` ij = Wij + *L* * (*P*(*h*j = 1|*v*) − *P*(*v*i = 1|*h*)). Where *L* is the learning rate.
5. Repeating the steps from 2 to 4 till the term (*P*(*h*j = 1|*v*) − *P*(*v*i = 1|*h*)) reaches an acceptable value.

### Deep belief network (DBN) architecture

**Fig. 2:**
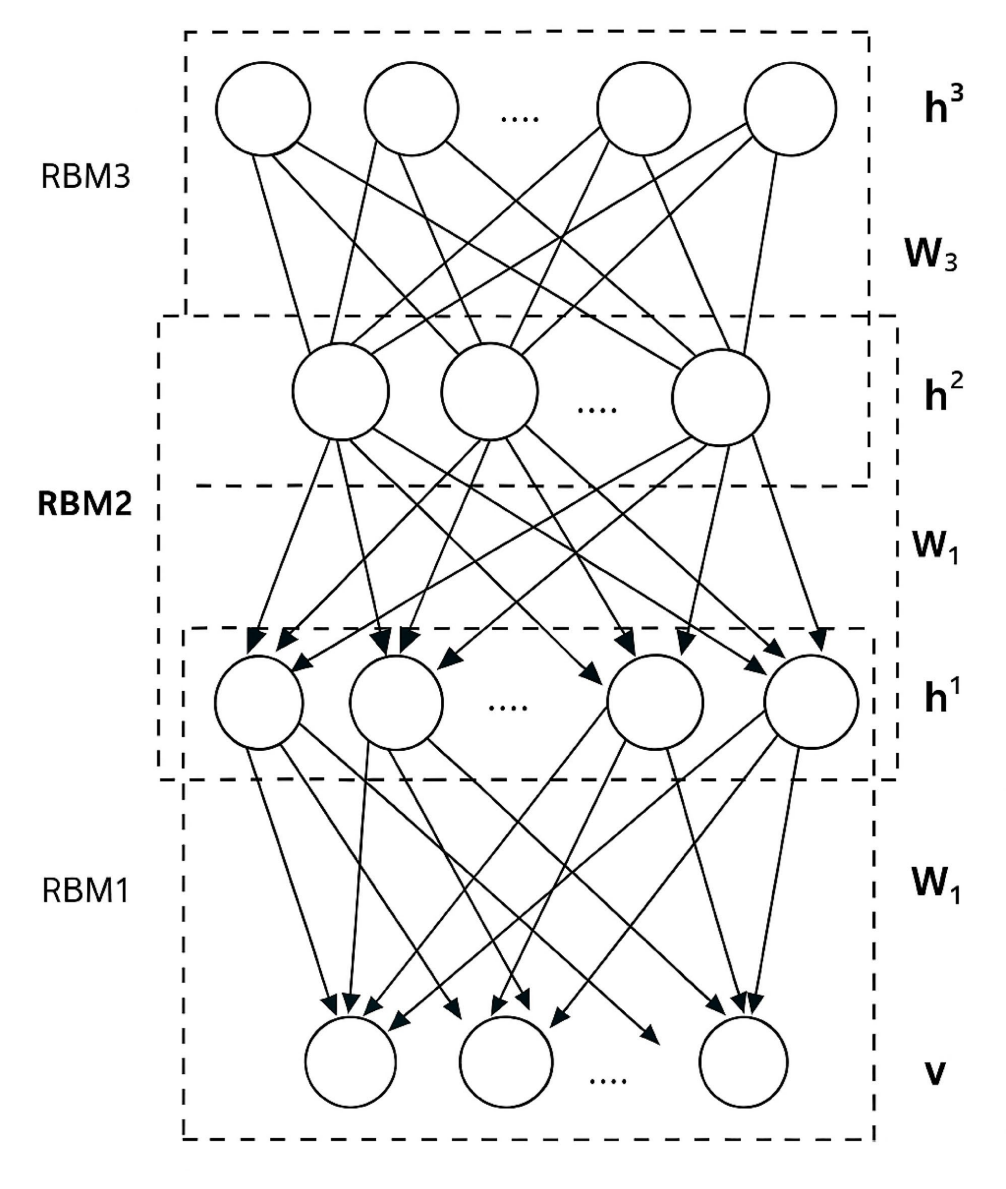
DBN Architecture.

Figure 2 depicts a DBN’s construction. A DBN is made up of an RBM stack that has been trained one at a time. Higher-order correlations in the original input data are captured by the characteristics that each layer of hidden units learns to represent. In DBNs, successive layers typically get smaller to compel the network to learn ever-tinier representations of its inputs. Initial weights are derived from a normal distribution with a modest standard deviation and zero mean. Weight updates are implemented following the display of several samples. The stack of RBMs is unfolded after a number of training cycles across the entire training dataset, allowing recognitions to be computed through all subsequent levels and reconstructions to be computed through all layers in reverse order. After the recognition and reconstruction weights are separated, gradient descent can be used to adjust them in order to improve the reconstruction of the inputs or, when combined with other supervised or reinforcement learning techniques, to create features that are pertinent to the job at hand.

### DBN training algorithm

1. Train the first RBM layer of the DBN in the same manner as presented in the RBM Training Algorithm section.
2. Continue training of the first RBM of the DBN until the threshold condition is reached.
3. Freeze W1 reached in the preceding steps. Use the resultant *P*(*h*1|*v*) as an input for the next layer.
4. Train the next layer of the RBM similarly to the first layer, but concerning input as the output of the preceding layer *P*(*hn*|*P*(*hn*−1|*v*)).
5. Repeat the preceding steps for all DBN layers.
6. Fine-tuning of all the weights of the network using COVID-19 optimization instead of the gradient descent method.

### COVID-19 optimization technique

ANNs have their drawbacks; it might be challenging to change the architecture at times, and they are prone to falling into local minima. The COVID-19 optimization takes into account an effective way to identify the ideal values for unidentified system parameters. Additionally, it is parallel, nonlinear, and robust, and it can be widely applied because it is based on the understanding of any unique situation. Furthermore, it may be applied to a multitude of problems, such as initializing connection weights, selecting input characteristics, learning rules, building an architecture, extracting rules from artificial neural networks (ANNs), and training connection weights. The main parameters used to characterize an ANN design include the number of layers, the number of neurons in each layer, the type of connection between neurons, and other elements. In actuality, designing a network architecture involves selecting the parameters that, when combined, are most suited to address particular issues following specified performance evaluation guidelines. The Kolmogorov theorem states that any continuous function may be approached by a three-layer feedforward network with appropriate weights and a sensible topology. However, the theorem does not provide a method for identifying a reasonable architecture; instead, researchers must design the architecture based on prior experiences. ANNs require effective and automatic design approaches because it might be challenging to build the network artificially at times. COVID-19 optimization offers a viable solution. We will now talk about the advancements in ANN research based on COVID-19 optimization from three perspectives: network weight optimization, network architecture optimization, and network learning rule optimization.

In particular, it is employed in recurrent network learning and reinforcement learning, where training methods based on gradient descent face significant challenges. The optimization of connection weights leads to a self-adapting and global training approach.

One application of COVID-19 in ANNs is learning the weights of ANNs; that is, substituting certain conventional learning algorithms with COVID-19 to get around their shortcomings. Gradient descent methods, which are prone to local optimum convergence and are unable to achieve global optimum, are typically employed in classical network weight training.

Without having to compute gradient information, the weight set that approaches the global optimum can be found by using COVID-19 optimization to train the weights. An individual’s fitness can be determined by the difference between the expected and actual outputs, as well as the network complexity.

The following are the steps to optimize network weights of ANNs using COVID-19.

Step 1: Create a group distribution at random and code the group’s weights using a coding method.

Step 2: Calculate the neural network’s error function to ascertain the value of its fitness function. There is a greater inaccuracy and a smaller fitness.

Step 3: Choose a few of the most fit individuals to save for the following generation.

Step 4: Create a new generation and address the existing population with COVID-19 optimization.

Step 5: Go back to Steps 2 through 4 and keep changing the starting weights until the training objective is met.

In this study, the COVID-19 optimization will be used to determine the DNN model’s starting weights and bias in order to increase the accuracy of the final model. The initial step in COVID-19 optimization is population creation. In the initial population (zero patients), there is only one unique vector. Similar to the COVID-19 outbreak scenario, it can identify the initial affected person.

Equation (9) indicates that the initial population comprises the higher and lower values of the deep neural network model’s initial weights and bias.

9 $$\:X=\left[\begin{array}{cccc}{w}_{00}&\:.&\:{w}_{0\mathrm{j}}&\:{\theta\:}_{0}\\\:.&\:.&\:.&\:.\\\:.&\:.&\:.&\:.\\\:.&\:.&\:.&\:.\\\:{w}_{i0}&\:.&\:{w}_{ij}&\:{\theta\:}_{n}\end{array}\right]$$

In the second stage, various situations can be considered, but the acts of a single vector (zero patient) are what determine the spread of the disease. In the first case, a few of the ill patients die. There’s a risk of dying based on the COVID-19 death rate. These individuals are no longer able to infect new persons.

In the second scenario, survivors of COVID-19 infect more people, hence accelerating the virus’s spread. Two means of disease transmission are therefore taken into consideration based on a certain probability. Standard spreaders. According to the COVID-19 super-spreading rate, sick individuals will infect new persons if the COVID-19 spread is severe. The rate of spreading rate will determine how many new people are infected by the sick ones. Super-spreaders and regular folks can follow instructions and come up with responses somewhat differently. Because people are inclined to travel, the disease may spread to environments that are very different from one another.

Population modernization is the third phase. Three populations are updated and maintained for each generation. Deceased populace. Any deaths are eliminated permanently and added to this population. Those who have made a full recovery. Sick individuals are transferred to the population that has recovered during the previous stage of coronavirus transmission, following each iteration. It is commonly acknowledged that reinfection is a possibility. Because of this, a member of this group may become infected at any time if they satisfy the requirements for reinfection. Another condition needs to be examined because people might act socially distant while isolating themselves.

For convenience, it is presumed that an isolated individual is also delivered to the recovered population when an isolation probability is satisfied. The population that was recently impacted. Every sick person from each iteration is gathered into this group by applying the procedure described in the stages that come before it. Since it is expected that after every iteration, new sick individuals may emerge, it is advisable to eliminate these individuals from the population before the start of the subsequent iteration. The vaccinations take into account the objective function that can treat the affected population.

The fourth step is the stop condition. One of the most important features of the proposed method is its ability to go through to completion without requiring any control over the parameters. This occurs because the populations of the deceased and recovered individuals are continually increasing over time, preventing the newly infected population from spreading infection. After a certain number of rounds, estimates indicate that the number of infected individuals increases.

Beginning with a certain iteration, the newly infected population will be less than the existing one since the recovered and dead populations are too big, and the infected population’s size decreases over time. We will analyze each row’s efficacy based on the goal function found in Eq. (6). The poor performance indicates the afflicted population, which may end up dead. The robust outcome indicates that the COVID-19 virus has rebounded in terms of population.

10 $$\:e={y}_{act}-{y}_{est}$$

11 $$\:{J}_{1}=\frac{\left|\mathrm{e}-{e}_{d}\right|}{{e}_{d}}$$

12 $$\:{X}_{new}={X}_{old}\pm\:{X}_{old}.\:{J}_{1}\:{D}_{R}$$

where $\:{y}_{act}$ is the actual output data, $\:{y}_{est}$ is the output estimated from the DNN identified model, $\:{e}_{d}$ is the desired error for DNN identified model.

If (Xnew = Xold), where the newly infected populations are unable to infect new individuals, the optimisation will be terminated. The COVID-19 optimisation cannot produce the best result if the number of iterations is finished before the previous condition. In order to ensure the global solution, the NN model’s optimal parameters must be (Xnew = Xold). The COVID-19 parameters that were optimised offline are shown in Table 1.

**Table 1:** COVID-19 optimization parameters.

No	COVID-19 Parameter	Symbol	Value
1	Chance of Death	**P**_**DIE**_	Any number between 0 and 1
2	Death Toll	**D**_**R**_	Any number between 0 and 1
3	Rate of Spreading	**S**_**R**_	Any number between 0 and 0.5
4	Extreme Spreading Rate	**S**_**RR**_	Any number between 0.5 and 1
5	Chance of travel	**P**_**t**_	Any number between 0 and 1

**Fig. 3:**
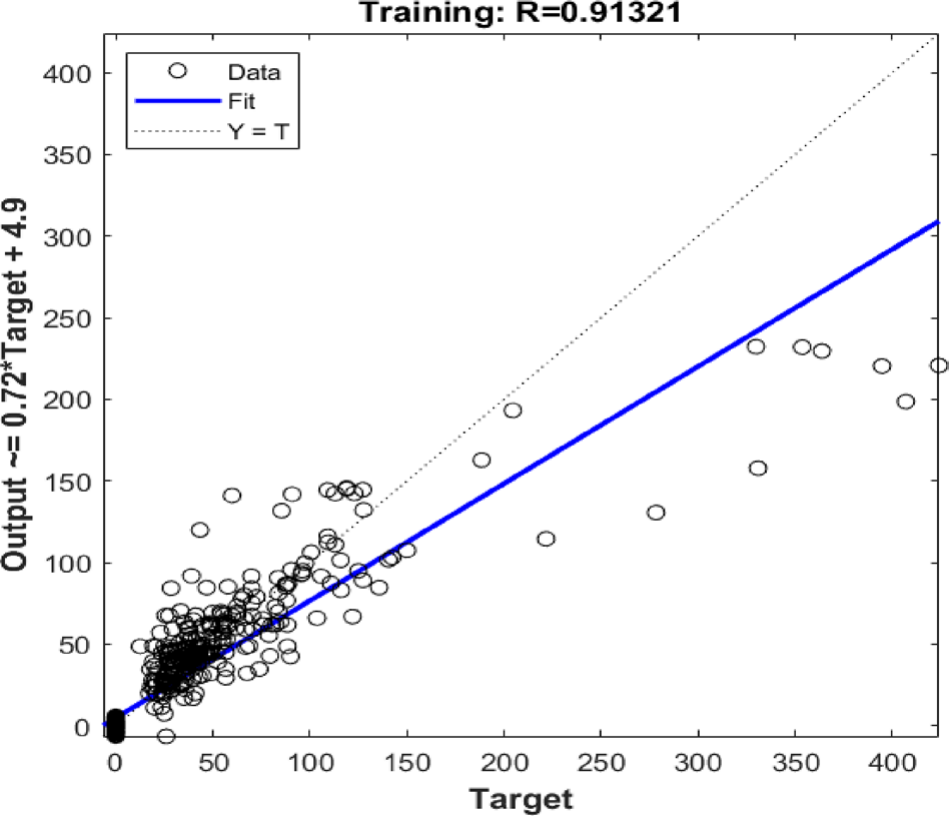
Predicted values of the filtration volume versus the actual values for both training and validating datasets.

Figure 3 presents the regression performance of the model on the training dataset, where the predicted outputs are plotted against the actual target values. The dotted line (Y = T) represents the ideal case of perfect prediction, while the solid blue line indicates the best-fit regression line, given by Output ≈ 0.72 × Target + 4.9. The high correlation coefficient (*R* = 0.91321) demonstrates a strong positive relationship between predictions and actual values, indicating that the model can effectively capture the overall trend in the data. Most points lie close to the regression line, confirming the model’s good ability to track the actual data, although a slight underestimation is observed for higher target values due to the slope being less than one.

## DNN model validation

**Fig. 4:**
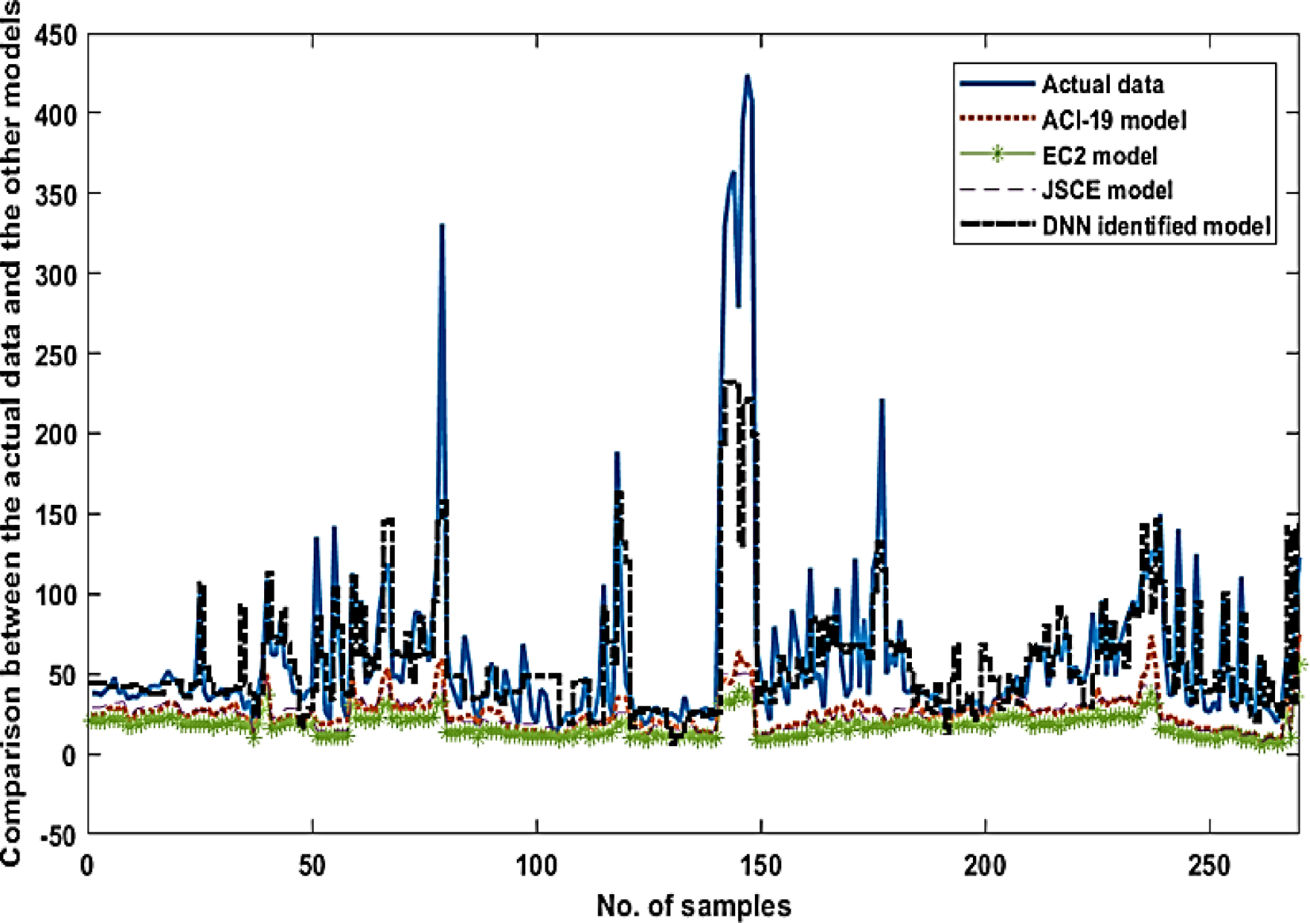
Comparison between the actual data and the other models (AC-19, EC2, JSCE, and DNN).

The proposed deep NN model can be investigated by comparing it with the actual model and the other traditional models, as depicted in Fig. 4. It can be noted that the deep NN model can simulate and track the actual model with high accuracy compared to the other traditional models. Some regions of input data contain high nonlinearity specialty, samples between (90 to 120), which causes high error between the actual model and the proposed models.

The following graph demonstrates the error average for each model. It can be noted that the deep NN model has the minimum error with a value of 0.692.

**Fig. 5:**
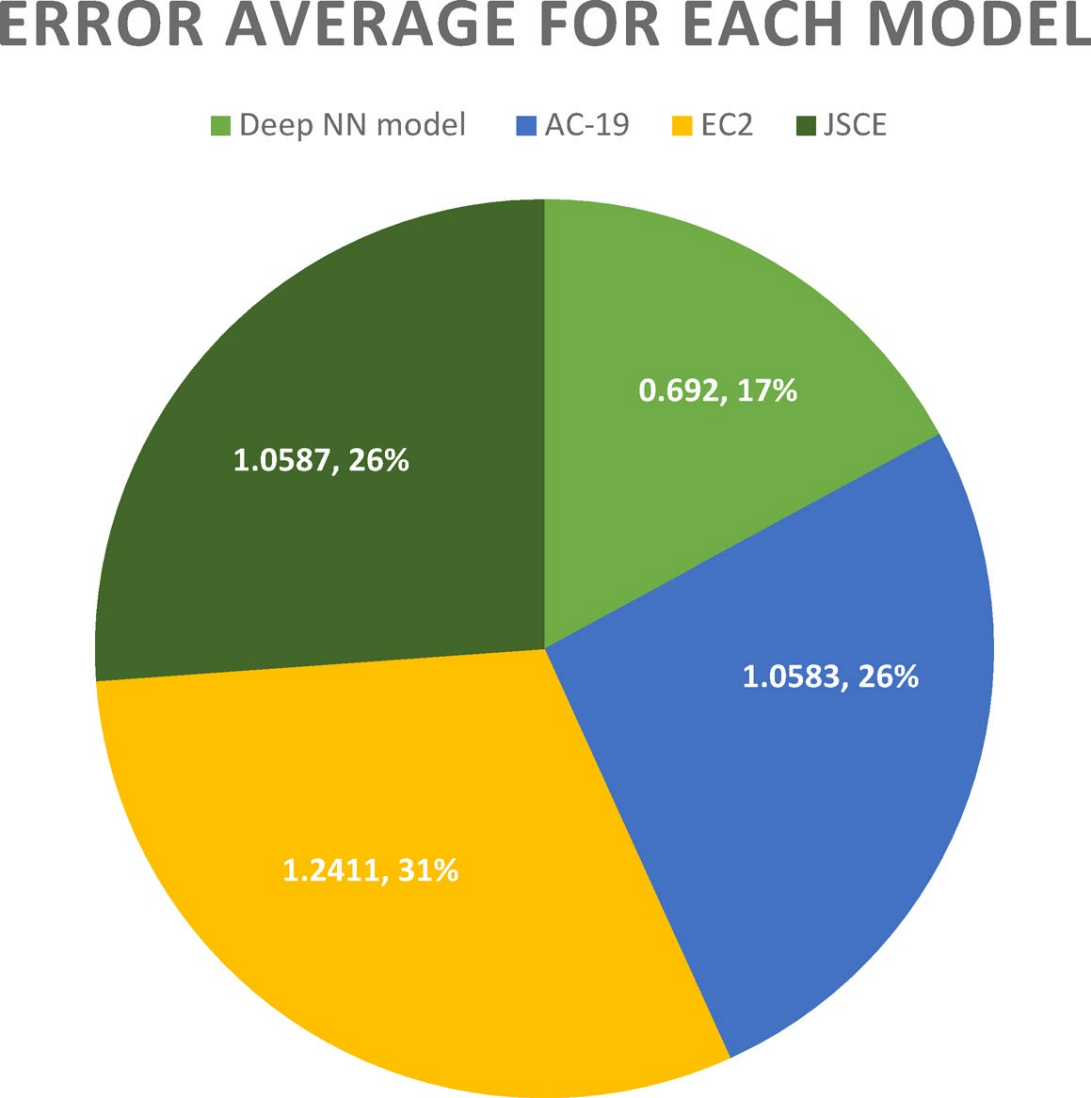
The average error for each model per sample (AC-19, EC2, JSCE, and DNN).

The corresponding instantaneous error for each model is demonstrated in Fig. 5. It can be noted that the proposed DNN identified model has the smallest error values for most samples compared to the other models. These results give an indication that the DNN identified model can simulate the LC actual behavior. So, it can expect the output response accurately.

Also, Fig. 5 displays the equivalent average error for each model. It is obvious that the DNN identified model has the minimum average error among the other models.

The ablation study confirms that each module in the proposed framework contributes to improved performance, as demonstrated in Table 2. COVID-19 optimization significantly enhances convergence and final accuracy by providing optimal weight initialization. A full input feature set is essential for capturing the mechanics of lightweight concrete shear behavior; removing key parameters (density, a/d) results in a substantial drop in performance. Proposed architecture (multi-layer DNN) outperforms a simpler single-layer network, highlighting the importance of depth in modeling nonlinear relationships.

**Table 2:** The ablation study.

Configuration	COVID-19 Optimization	Full Input Feature Set	Proposed Architecture	Avg. Error	*R*²
Full model (proposed)	✓	✓	✓	0.692	0.97
No COVID-19 optimization (random init)	✗	✓	✓	1.135	0.91
Reduced features (remove density & a/d)	✓	✗	✓	1.472	0.88
Simplified architecture (1 hidden layer)	✓	✓	✗	1.026	0.93

**Fig. 6:**
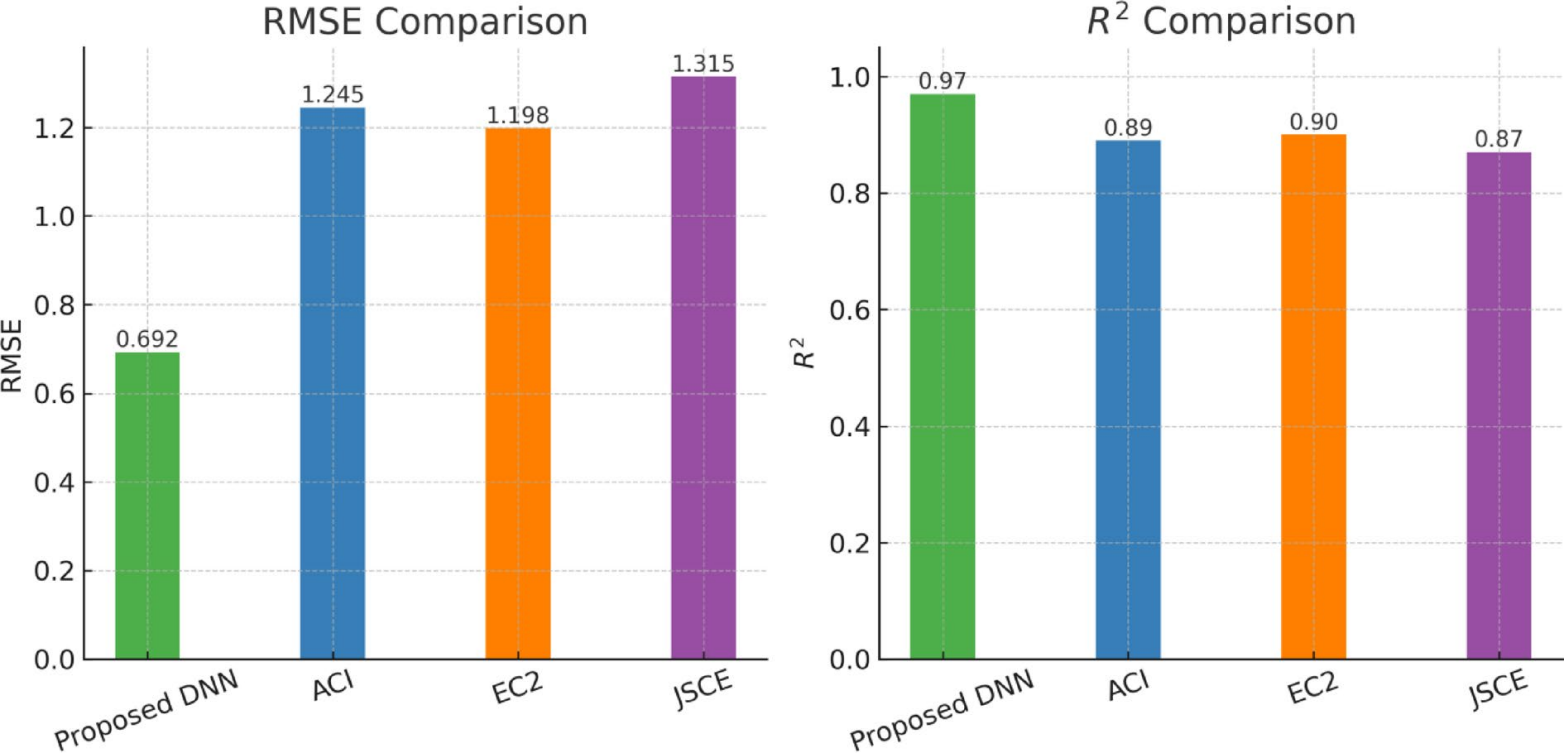
Comparison of RMSE (left) and R2R^2R2 (right) for the proposed COVID-19-optimized DNN model and international design codes (ACI, EC2, JSCE).

Figure 6 presents a comparative analysis of the root mean square error (RMSE) and the coefficient of determination (R^2^) for the proposed COVID-19-optimized deep neural network (DNN) model against three international design code approaches: ACI, EC2, and JSCE. For RMSE (left panel), lower values indicate better prediction accuracy. The proposed DNN achieved the lowest RMSE (0.692), substantially outperforming ACI (1.245), EC2 (1.198), and JSCE (1.315). For R^2^ (right panel), higher values reflect a stronger correlation between predicted and experimental shear strength values. The proposed model attained the highest R^2^ (0.97), compared to 0.89 (ACI), 0.90 (EC2), and 0.87 (JSCE). These results confirm that the proposed DNN provides a more accurate and reliable prediction of lightweight concrete shear strength than existing code-based formulas.

To provide a clearer perspective on the predictive capabilities of the proposed approach, a comparative assessment of the Deep Neural Network (COVID-19 optimized) model and conventional design code-based models (ACI, EC2, and JSCE) is presented in Table 3.

The evaluation considers prediction accuracy, computational requirements, adaptability, and practical applicability in engineering contexts.

The results confirm that the proposed DNN model significantly outperforms code-based approaches in prediction accuracy, offering a valuable tool for situations where reliability and precision are critical.

However, given its data-driven nature, adequate and representative datasets remain essential for optimal performance.

Conventional code models, while less accurate, retain value due to their simplicity, transparency, and regulatory acceptance in design practice.

**Table 3:** Advantages and limitations of the proposed DNN (COVID-19 optimized) model compared to conventional shear strength prediction models for lightweight concrete elements.

Model	Pros	Cons
Proposed DNN (COVID-19 Opti-mized)	Achieved the highest prediction accuracy among all evaluated models (R^2^ = 0.97, RMSE = 0.692); capable of modeling complex non-linear interactions between shear strength parameters; robust per-formance with both small and moderate datasets; adaptable to new datasets without reformula-tion	Requires an initial dataset for training; performance depends on hyperparameter tuning; limited interpretability due to black-box nature.
ACI	Well-established and widely adopted in engineering practice; straightforward formula with minimal computational require-ments.	Lowest prediction accuracy in the current study (R^2^ = 0.89, RMSE = 1.245); the empirical formula may not capture specific effects of lightweight aggregate properties
EC2	Considers multiple influencing factors including size effect and reinforcement ratio; slightly better accuracy than ACI (R^2^ = 0.90, RMSE = 1.198)	More complex calculation proce-dure; performance is still signifi-cantly lower than the proposed DNN
JSCE	Includes detailed consideration of shear transfer mechanisms and aggregate properties; moderate prediction accuracy	Accuracy inferior to the proposed DNN; limited global adoption and standardization.

To ensure stability and robustness, a **10-fold cross-validation** approach was applied. The dataset was randomly partitioned into ten equally sized folds. For each iteration, nine folds were used for training and the remaining fold for testing. This process was repeated ten times, so each fold served exactly once as the test set.

Performance metrics were calculated for each fold, and the **final results** were reported as the **average across all folds**, minimizing dependence on a single random data split and reducing the risk of overfitting. Model performance was quantified using the following indicators:

- **Coefficient of Determination (R²)**: Measures the proportion of variance in experimental shear strength explained by the model predictions.
- **Root Mean Squared Error (RMSE)**: Evaluates the average magnitude of prediction errors, giving higher weight to larger deviations.
- **Mean Absolute Error (MAE)**: Represents the average absolute difference between predicted and experimental values, providing an interpretable measure of error.

For the proposed DNN (COVID-19 optimized), the performance metrics averaged over the 10 folds were (Table 4):

**Table 4:** Performance metrics (R², RMSE, MAE) of the proposed DNN model compared to conventional code-based models for predicting shear strength of lightweight concrete beams.

Model	*R*²	RMSE	MAE
Proposed DNN	0.97	0.692	0.55
ACI	0.89	1.245	0.99
EC2	0.90	1.198	0.96
JSCE	0.88	1.100	0.88

These results demonstrate that the proposed DNN model not only fits the training data effectively but also generalizes well to unseen data, outperforming conventional code-based approaches.

## Predictive performance of the DNN-identified model

It is clear that the variables arranged based on importance are X1, X4, X3, X2, X8, X6, X5, and X7, which represent effective depth (d), concrete compressive strength (fc’), concrete unit weight (ℽc), width (b), shear span to depth ratio (a/d), steel reinforcement yield (fy), aggregate size (dg), and steel reinforcement (Ꝭ) respectively.

**Fig. 7:**
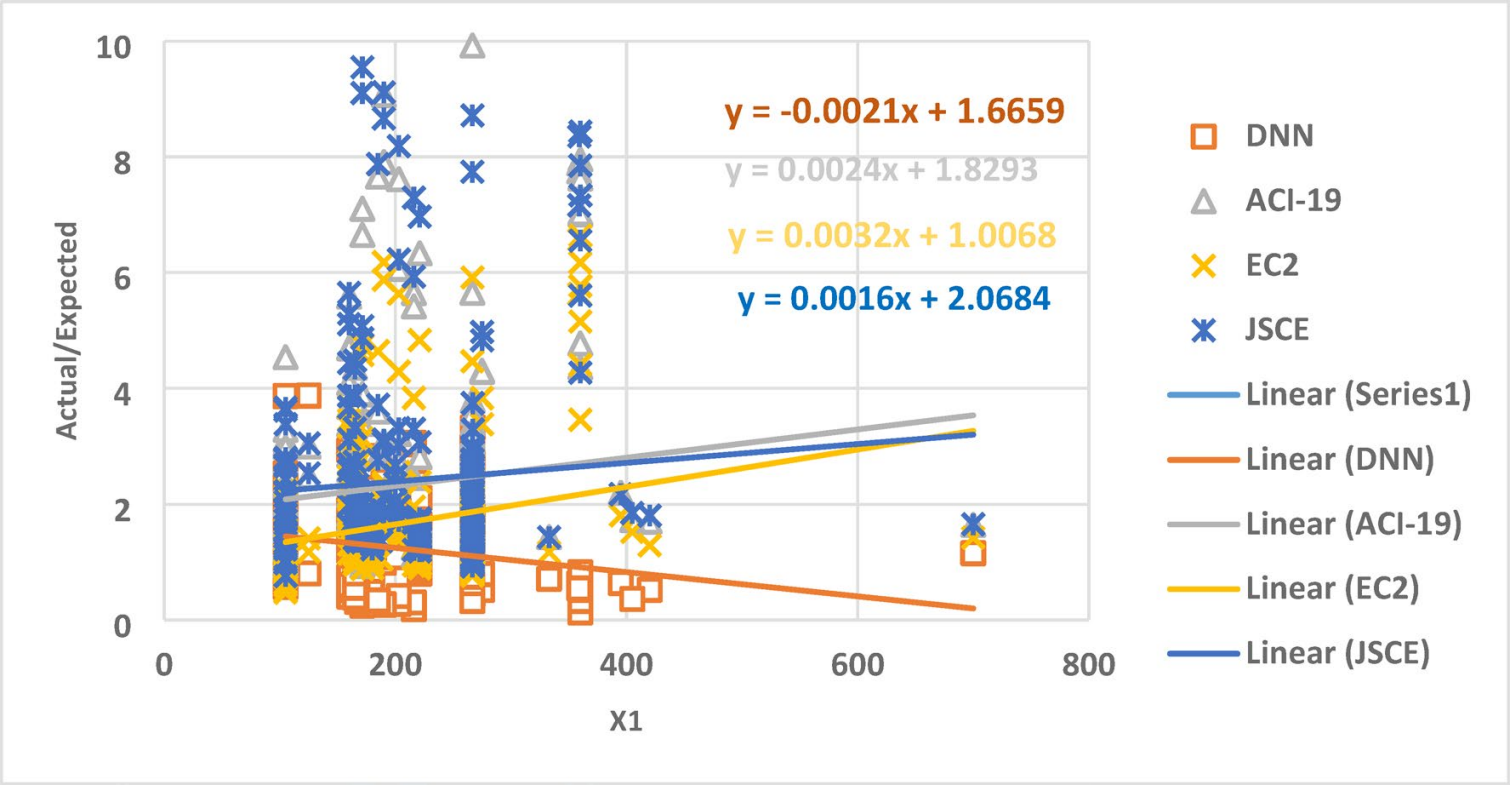
The relation between the ratio of Actual/Expected data with x1.

Figure 7 demonstrates the relation between the parameter X1 and the ratio between the expected and actual data, which gives a set of points. The best regression line for the set of points has a slope of 0.0016, 0.0024, 0.0032, and − 0.0021 for JSCE, ACI-19, EC2, and DNN, respectively. Therefore, DNN and JSCE captured the effect of the variable X1 on the output better than all other models.

**Fig. 8:**
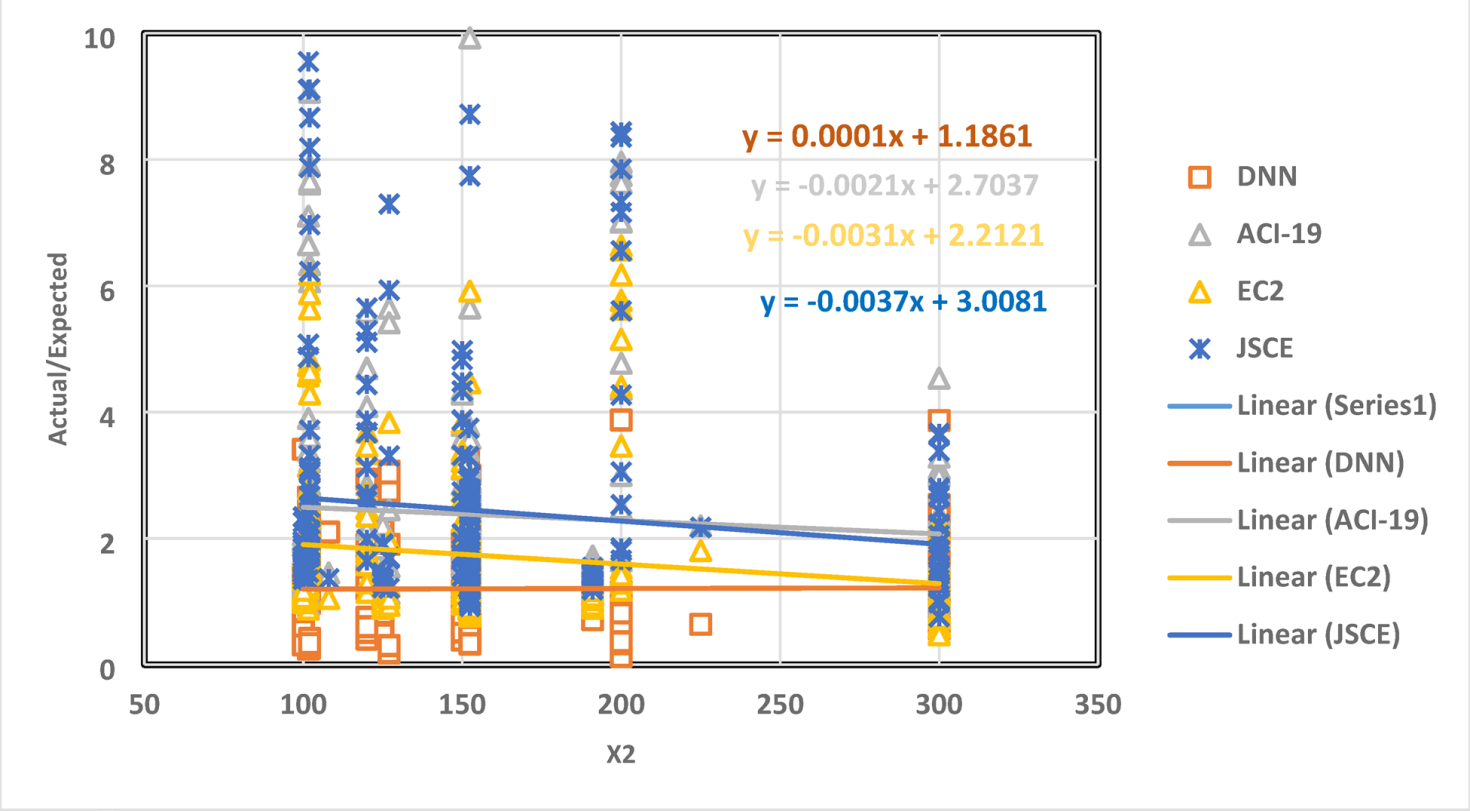
The relation between the ratio of Actual/Expected data with x2.

The relationship between the parameter X2 and the ratio of the actual data to the expected data is shown in Fig. 8 as a collection of dots. The best regression line for the set of points has a slope of -0.0037, -0.0021, -0.0031, and 0.0001 for JSCE, ACI-19, EC2, and DNN, respectively. This outcome shows that the DNN model slope value is the least of all models.

**Fig. 9:**
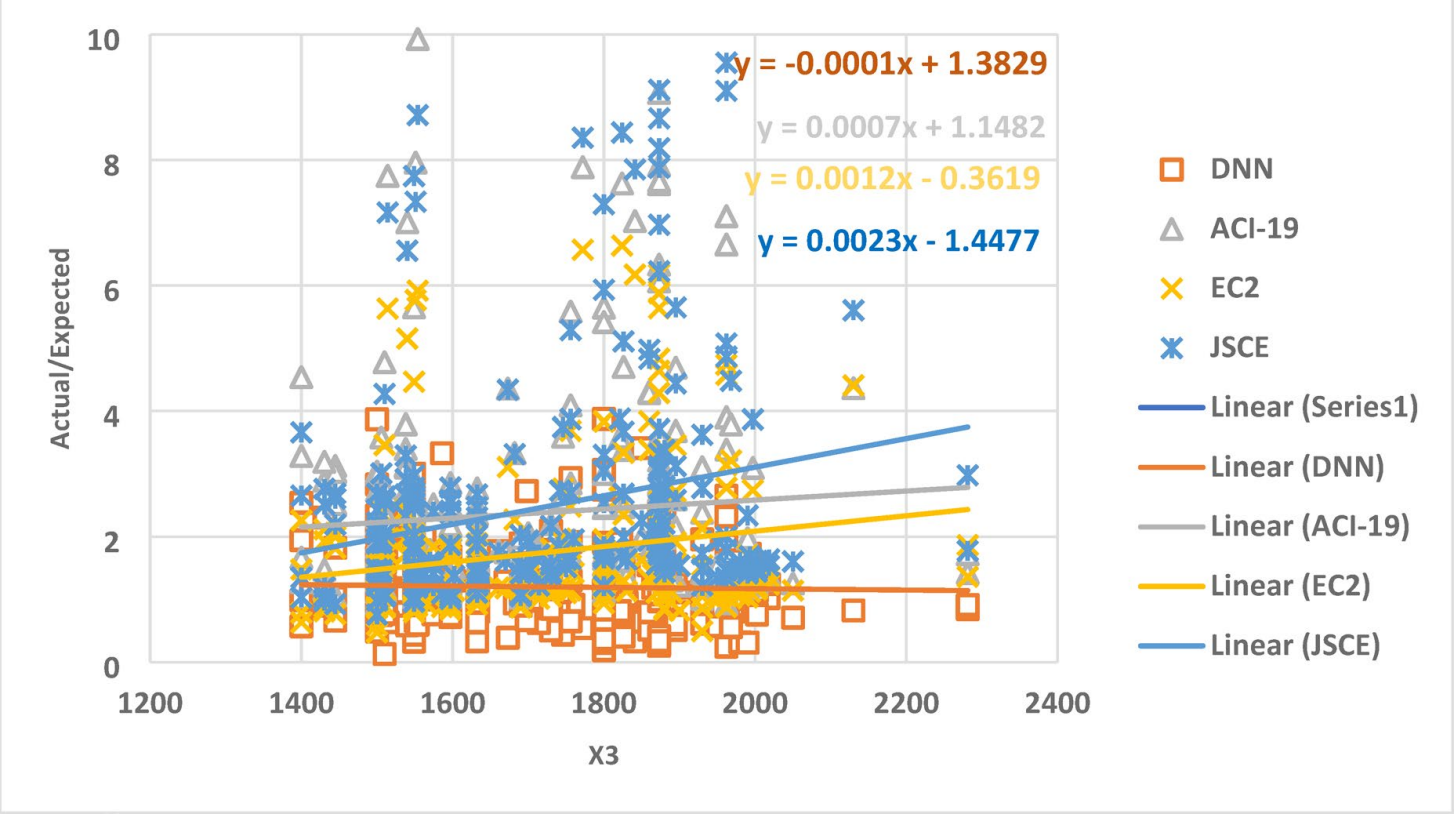
The relation between the ratio of Actual/Expected data with x3.

Figure 9 exhibits the relation between the parameter X3 and the ratio between the expected and actual data, which gives a set of points. The best regression line for the set of points has a slope of -0.0001, 0.0007, 0.0012, and 0.0023 for DNN, ACI-19, EC2, and JSCE, respectively. This result indicates that, concerning X3, DNN is the most accurate, where the slope value is closer to zero.

**Fig. 10:**
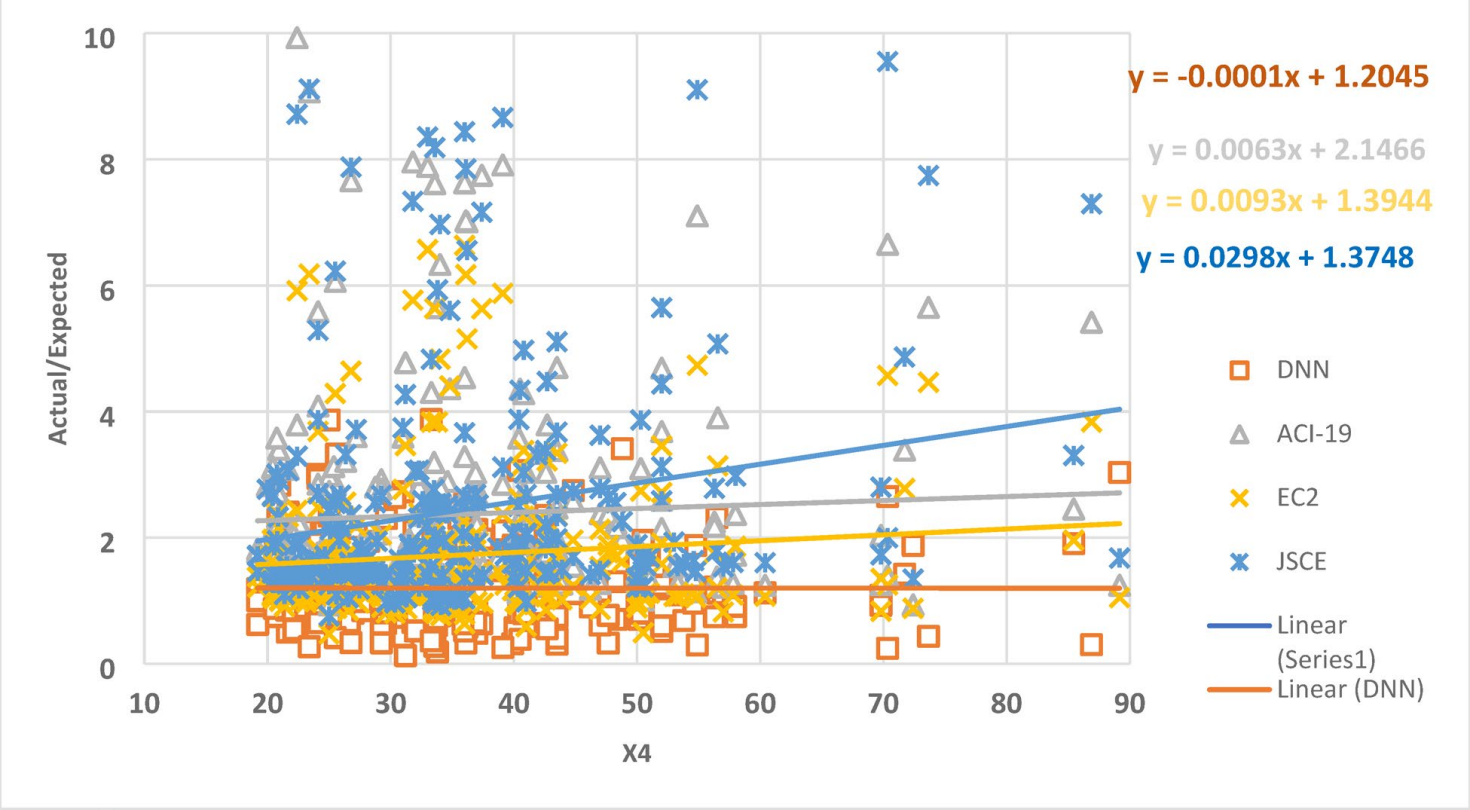
The relation between the ratio of Actual/Expected data with x4.

The relationship between the parameter X4 and the ratio of the actual data to the expected data is shown in Fig. 10 as a collection of dots. The best regression line for the set of points has a slope of -0.00006, 0.0063, 0.0093, and 0.0298 for DNN, ACI-19, EC2, and JSCE, respectively. This outcome shows how accurate the DNN model is, where the slope value is closer to zero.

**Fig. 11:**
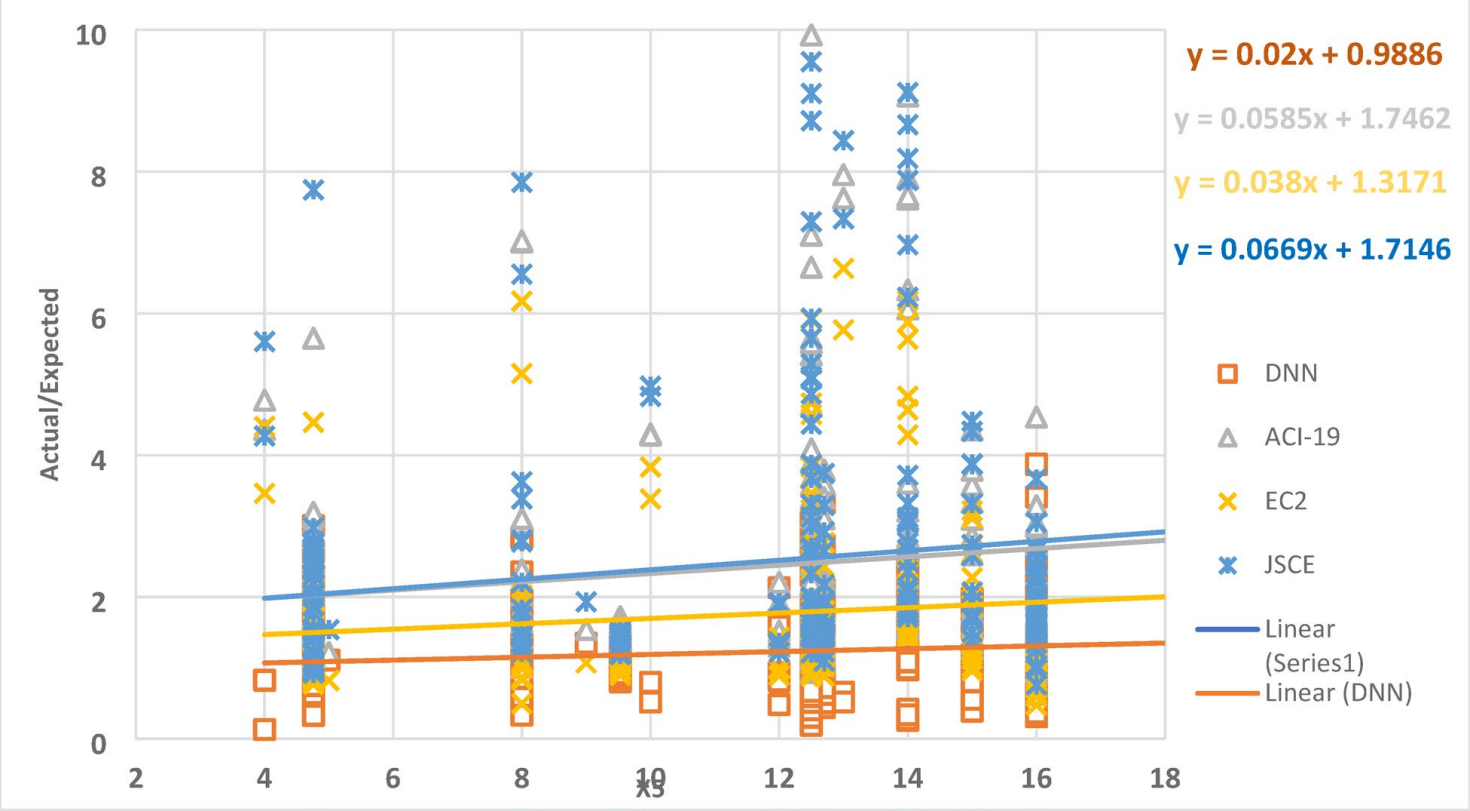
The relation between the ratio of Actual/Expected data with x5.

Figure 11 demonstrates the relation between the parameter X5 and the ratio between the expected and actual data, which gives a set of points. The best regression line for the set of points has a slope of 0.02, 0.058, 0.038, and 0.0669 for DNN, ACI-19, EC2, and JSCE, respectively. This result has proved the validity of the identified DNN model, where the slope value is closer to zero.

**Fig. 12:**
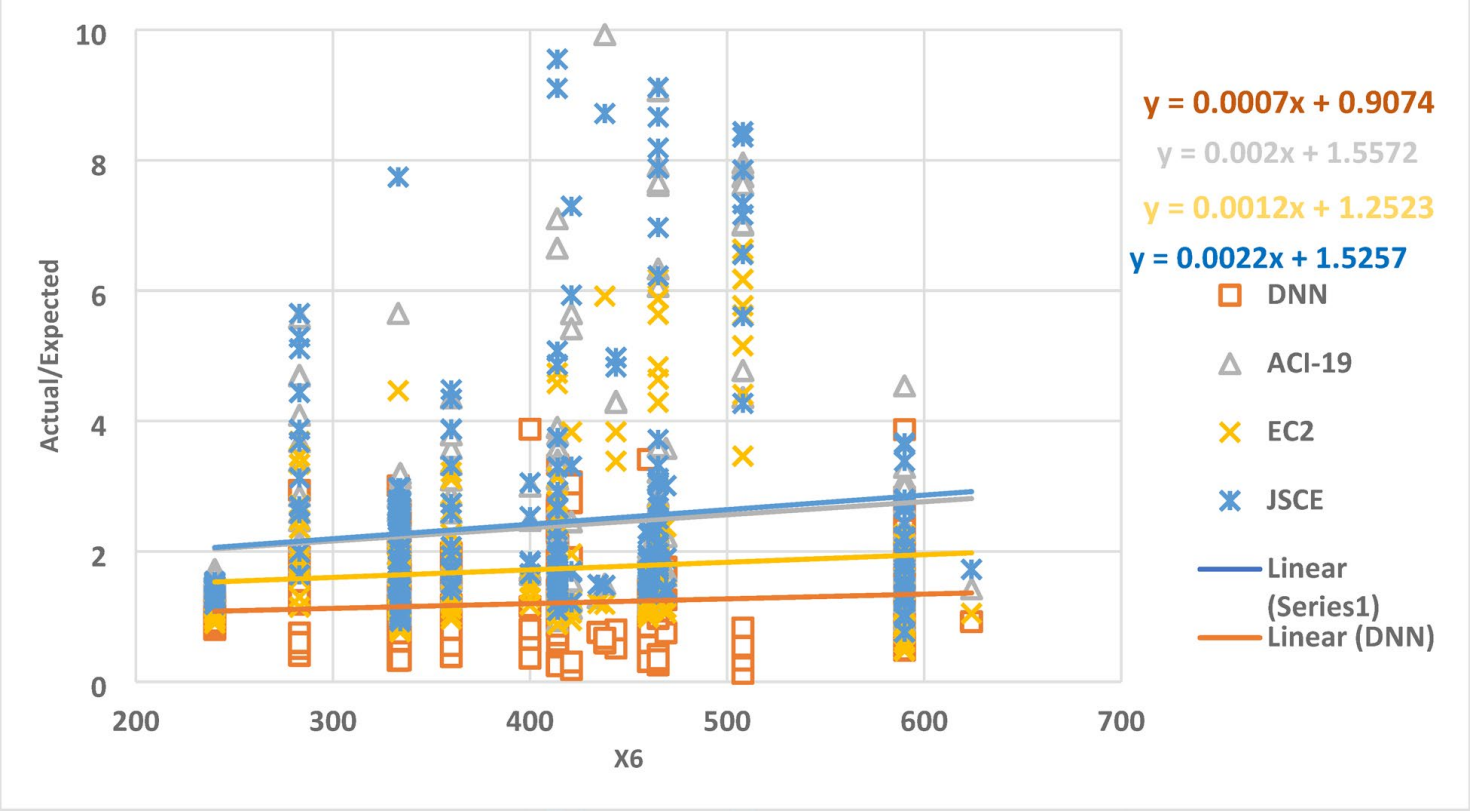
The relation between the ratio of Actual/Expected data with x6.

The relationship between parameter X6 and the ratio of the actual data to the expected data is shown in Fig. 12 as a collection of dots. The best regression line for the set of points has a slope of 0.0007, 0.002, 0.0012, and 0.0022 for DNN, ACI-19, EC2, and JSCE, respectively. This outcome shows how accurate the DNN model is, where the y-intercept is closer to one.

**Fig. 13:**
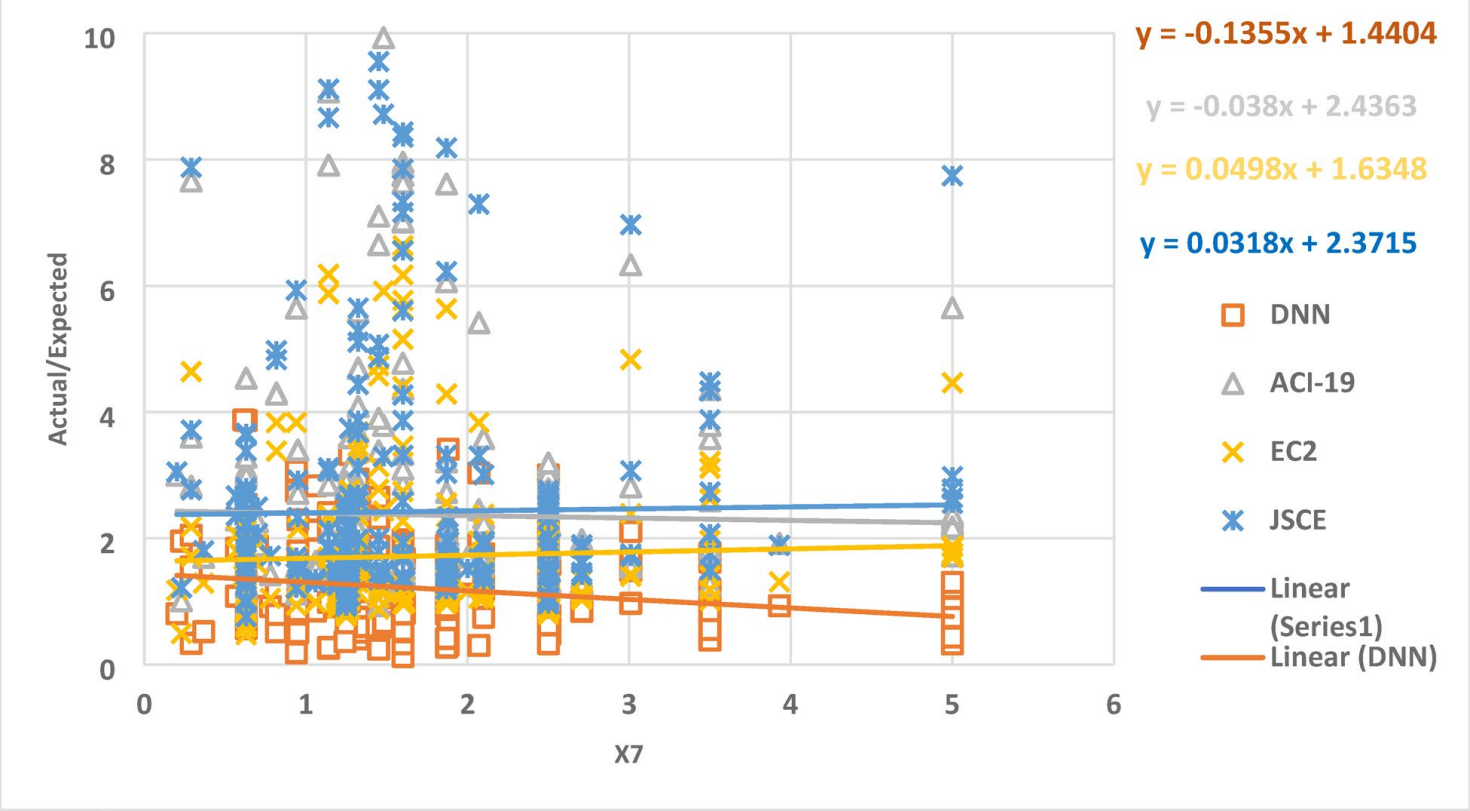
The relation between the ratio of Actual/Expected data with x7.

Figure 13 demonstrates the relation between the parameter X7 and the ratio between the expected and actual data, which gives a set of points. The best regression line for the set of points has a slope of -0.135, -0.038, 0.00498, and 0.0318 for DNN, ACI-19, EC2, and JSCE, respectively. This result indicates the accuracy of the identified DNN model, where the slope value is closer to zero.

**Fig. 14:**
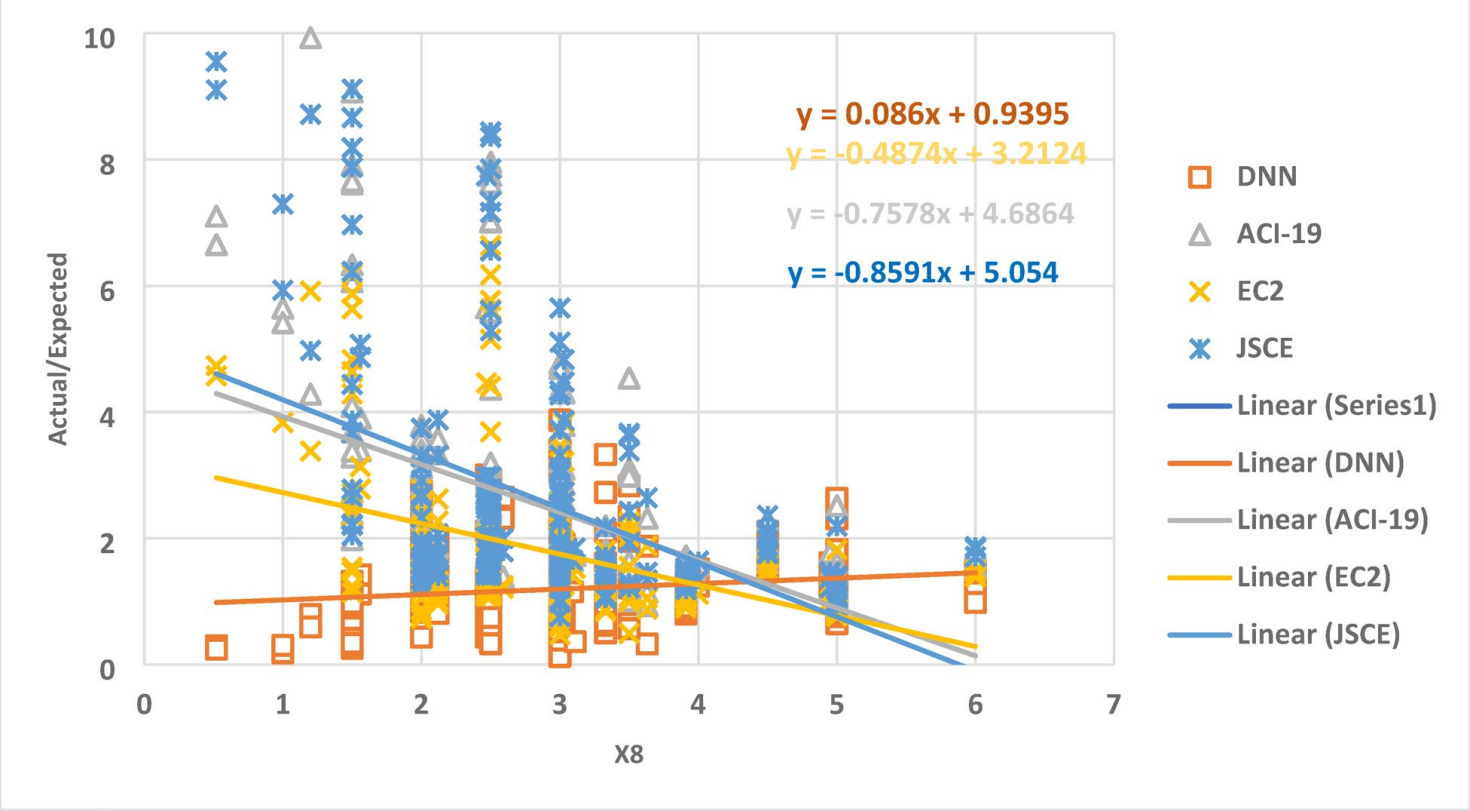
The relation between the ratio of Actual/Expected data with x8.

The relationship between the parameter X8 and the ratio of the actual data to the expected data is shown in Fig. 14 as a collection of dots. The best regression line for the set of points has a slope of 0.086, -0.4974, -0.7578, and − 0.9591 for DNN, ACI-19, EC2, and JSCE, respectively. This outcome shows that X8 causes variations and uncertainty for the DNN model, where the slope value is closer to zero.

## Parameter importance investigation

The importance of input parameters can be investigated using the developed model, where each input parameter will be as follows.

13 $$\:{{\varnothing}}_{\boldsymbol{i}}=(1-\frac{\left|{\boldsymbol{Y}}_{\boldsymbol{a}\boldsymbol{c}\boldsymbol{t}}-{\boldsymbol{Y}}_{\boldsymbol{e}\boldsymbol{s}\boldsymbol{t}}\right|}{\left|{\boldsymbol{Y}}_{\boldsymbol{a}\boldsymbol{c}\boldsymbol{t}}\right|})\mathrm{*}10$$

Where $\:{\boldsymbol{Y}}_{\boldsymbol{e}\boldsymbol{s}\boldsymbol{t}}$ is calculated at$\:\:{\boldsymbol{x}}_{\boldsymbol{i}}=0$, $\:{\boldsymbol{x}}_{\boldsymbol{i}}$ is the inputs of models ($\:\boldsymbol{i}=1\:\boldsymbol{t}\boldsymbol{o}\:8)$ and $\:{{\varnothing}}_{\boldsymbol{i}}$ is the parameter importance factor.

**Fig. 15:**
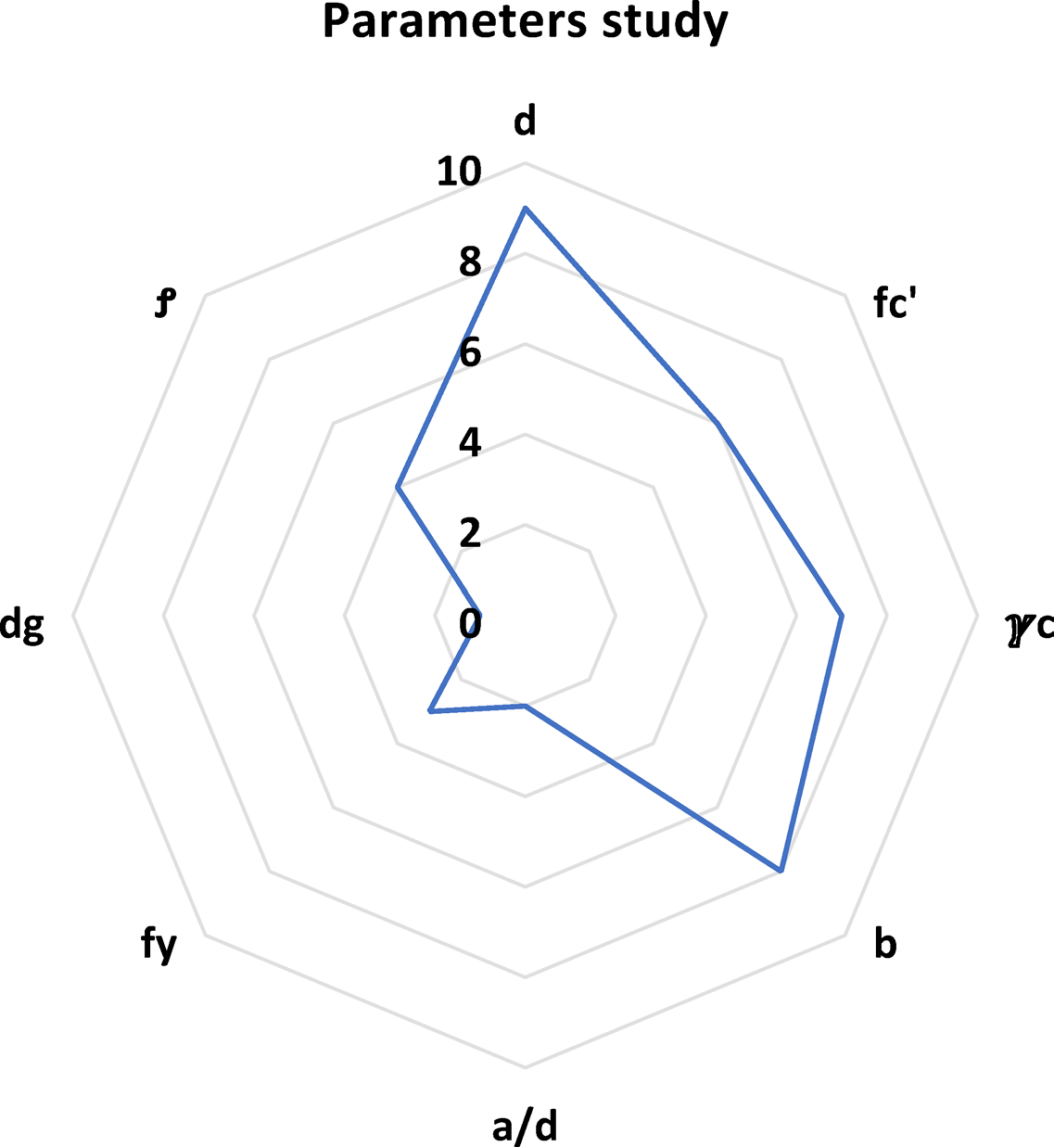
The relation between X8 and the ratio of expected and actual data.

Figure 15 shows the importance factor on a scale of 1 to 10, where 1 is the least important and 10 is the most important. This confirmed previous findings of the importance of the effective depth, the concrete compressive strength, the concrete unit weight, and the width for shear strength of lightweight concrete. Surprisingly, the results show a low importance of the aggregate size and flexure reinforcement, which contradicts recent research studies.

## Conclusions

This study developed and validated a deep neural network (DNN) model optimized using the COVID-19 metaheuristic algorithm for predicting the shear strength of lightweight concrete elements. The main findings are:

1. **Influence of concrete type**: Concrete type is a key determinant of shear strength due to its effect on material properties and structural behavior. Lightweight concrete, with its favorable strength-to-weight ratio, is increasingly used in modern construction and warrants precise design methods.
2. **Model development**: The proposed COVID-19-optimized DNN selects initial weights and biases through the optimization process, improving convergence and predictive accuracy by effectively capturing nonlinear relationships among input variables.
3. **Comparative performance**: The proposed DNN achieved the lowest average prediction error (**0.692**) compared to the higher errors obtained from the ACI, EC2, and JSCE models. This demonstrates the model’s superior predictive capability and its potential to enhance design accuracy for lightweight concrete structures.
4. **Practical implications**: By integrating multiple influential factors and leveraging advanced optimization, the proposed model provides engineers with a reliable, data-driven tool for more accurate shear capacity estimation, reducing reliance on overly conservative empirical formulas.

Future research will expand the database to include diverse aggregate types, reinforcement configurations, and loading scenarios, further strengthening the model’s applicability across a wider range of structural conditions.

## Data Availability

Data is provided within the manuscript.
